# A General Kinematic Model for Multimodal Locomotion in Bioinspired Robots

**DOI:** 10.34133/research.1275

**Published:** 2026-05-04

**Authors:** Zicun Hong, Junwen Fei, Weihua Li, Jun Yan, Junzhi Yu, Feng Tian, Yong Zhong

**Affiliations:** ^1^ Shien-Ming Wu School of Intelligent Engineering, South China University of Technology, Guangzhou, China.; ^2^ GRG Banking Equipment Co. Ltd., Guangzhou, China.; ^3^ Chinese Institute of Marine & Offshore (ZH) Co. Ltd., Zhuhai, China.; ^4^ State Key Laboratory for Turbulence and Complex Systems, Department of Advanced Manufacturing and Robotics, College of Engineering, Peking University, Beijing, China.

## Abstract

Locomotion in animals such as fish, snakes, inchworms, and octopuses exhibits a remarkable diversity, with each species utilizing distinct body morphologies and movement strategies. Currently, no existing kinematic model is capable of describing the full range of locomotion exhibited by these animals. Addressing this challenge holds important implications for both the study of biomechanics of animals and the development of bioinspired robots. In this work, we propose a general kinematic model that integrates the curvature equation with a nonlinear oscillator. Through parameter adjustments, its morphology can transition between the motions of various animals. It is the most versatile kinematic model to date for describing multimodal locomotion of animals so far as we know. By translating the general kinematic model into a motion control algorithm and combining it with virtual simulation, we create a motion optimization framework that substantially simplifies the complexity of multimodal control for bionic robots with diverse actuation mechanisms, thereby enhancing their maneuverability. Using fish locomotion as an example, we validate the methodology on an untethered multijoint robotic fish, successfully enabling the robotic fish to perform cruising and various fast turn motions, thereby demonstrating its effectiveness in guiding motion control. This work is believed to have laid the foundation for the study of bionic motion and bioinspired robots.

## Introduction

**Fig. 1.**
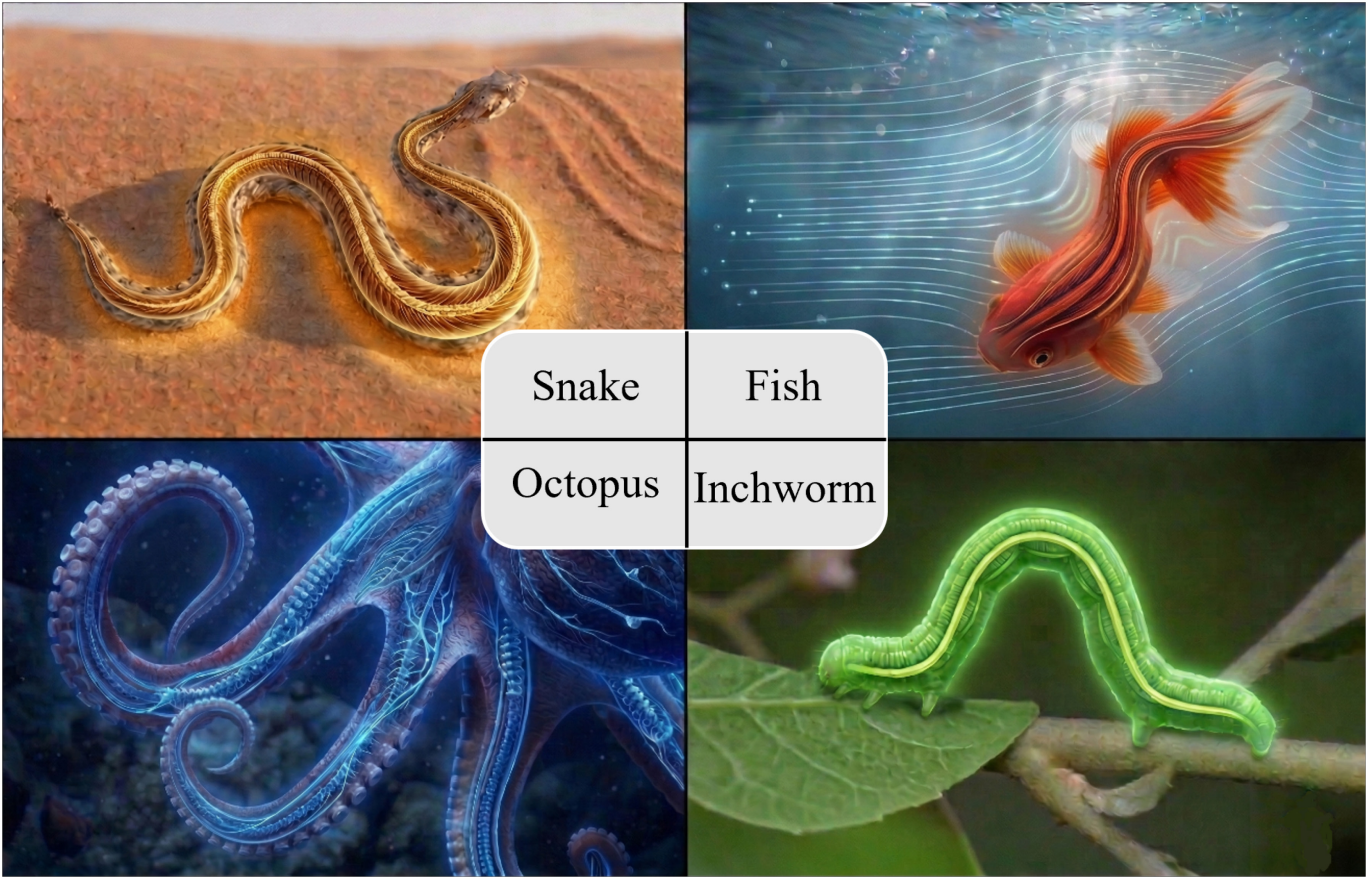
Various animals that move by bending their bodies.

Numerous species in the nature are capable of generating a wide variety of locomotions through the bending of their bodies (Fig. 1), which allows them to adapt to complex environmental conditions, evade predators, and hunt more effectively. To study these animals in depth, many researchers have focused on understanding their motions, aiming to uncover the underlying mechanisms and inspire advances in the field of bionics. Initially, researchers had to capture numerous images of the animal’s movements and then extract the body’s midline through image-fitting techniques [1–4], serving as the foundation for motion analysis.

If the species or motion types change, this process must be repeated, making it highly time-consuming. Therefore, constructing corresponding kinematic models tailored to the motion characteristics of different species could considerably enhance the efficiency of motion analysis [5]. The limbless locomotion of snakes has attracted considerable attention due to their highly adaptable motions, enabling them to move rapidly across land, water, and in confined spaces [6,7]. To facilitate biomechanical research on snakes, Hirose [8] proposed a serpenoid curve that mimics their most common serpentine movement. Ma [9] further refined this approach by proposing a more efficient serpentine curve that accounts for the snake’s muscular characteristics. By adjusting parameters, these curves can describe various motions of snakes, such as straight and turning. On this basis, a number of snake like robots have been developed [10–12]. The inchworm motion is another archetypal rhythmic motion, characterized by the formation of an “Ω” posture during periodic alternations between bending and stretching phases to achieve crawling. Using body curvature as a variable, several models have been proposed to describe the crawling, climbing, and transitioning locomotion of inchworms [13,14], thus accelerating the development of robotic inchworms [15,16]. Octopus soft arms exhibit similar characteristics, with infinite degrees of freedom allowing them to bend flexibly and extensively, enabling bend propagation motions for prey capture. To model it, Xie et al. [17] introduced the classical “rose line” equation in polar coordinates and further divided it into 2 types of motion: reaching and sweeping, which has been used to guide the design of bionic soft arms.

In addition to the aforementioned species, fish swimming in body-caudal fin mode are also typical examples of using their bodies for propulsion, exhibiting a wide variety of motions. Their motions can be classified into 2 primary categories based on their motion characteristics: cruising and fast turns. Cruising refers to steady-state locomotion, which includes cruising straight and cruising turn, where the fish swims at a nearly constant velocity in a straight line or during a turn, typically occurring over longer distances. In contrast, fast turns are explosive motions, often completed within a fraction of a second, in which the fish rapidly alters its body posture to change its direction of travel. These motions are primarily observed during predation or escape behaviors [18]. During fast turns, fish bend their bodies into “C” or “S” shapes, referred to as C-turns or S-turns, respectively. These diverse motions are key to the fish’s ability to adapt to the complex underwater environment. To develop a new generation of underwater robots, scientists have proposed many robotic fish prototypes and associated technologies [19–22]. For robotic fish that mimic natural fish as an object, a kinematic model of fish’s body midline has important guiding significance in motion control. The traveling wave equation proposed by Lighthill [23] remains the most widely accepted kinematic model for fish locomotion. Composed of an envelope equation and a sine function, this model uses the lateral displacement of the fish body as the dependent variable and effectively describes the body midline morphology during cruising-straight motion. However, the model’s limited ability to account for large curvature and its inherent geometric and temporal symmetries prevent it from accurately modeling fish turning motions. To address this limitation, Liu and Hu [24,25] divided the fish body midline into 2 secondary curves, developing a kinematic model resembling a semicircle to describe the C-turn. This model successfully guided robotic fish in executing the corresponding motion. In addition, several researchers have attempted to construct kinematic models using body curvature as the dependent variable, which increases the model’s bending capacity and better represents the body morphology during turns. Gazzola et al. [26] incorporated a baseline offset and a specific time-varying function into a curvature-based model, breaking its symmetry in both geometry and time to enable the description of C-turns. Similarly, Leroyer and Visonneau [27] adopted a curvature-based modeling approach, introducing a tunable baseline offset that allowed the model to transition between cruising-straight and C-turn motion. Although several new models have been proposed in recent years [28,29], none of them has been able to describe all morphologies in both cruising and fast turns, complicating the multimodal control of robotic fish.

To resolve this issue, based on the central pattern generator (CPG) model in [30,31], our team has integrated a nonlinear oscillator with the traveling wave equation, creating a general kinematic model capable of illustrating fish multiple swimming motions [32]. Unlike traditional CPG methods that often entangle temporal rhythms with spatial joint angle mappings, it strictly decouples these 2 domains. By utilizing a nonlinear oscillator exclusively for rhythm generation while governing the spatial morphology through traveling wave equation, the proposed model achieves superior motion precision and enables seamless transitions between distinct locomotion modes. However, as the model is based on the traveling wave equation, it struggles to describe large curvature motions, such as a C-turn that exceeds a semicircle in curvature, limiting its ability to capture sharp fast turns with extremely high turning angles. However, the ability to perform sharp fast turns is crucial for achieving high maneuverability in fish. In addition, this shortcoming also limits its potential for describing the motions of large curvature animals such as snakes, inchworms, and octopus arms.

For serial bionic robots such as multijoint robotic fish, the trajectory approximation method is a widely used algorithm in motion control, which works by fitting the body midline of the animal through coordinated control of the driving joints to achieve locomotion [33]. If there is a kinematic model of the biological object’s body midline as a reference benchmark, this algorithm can be implemented efficiently. Otherwise, it is necessary to customize the angle of each joint, which is not only cumbersome but also reduces the accuracy of motion control, particularly for fast, large-amplitude motions [34,35]. Therefore, developing a general kinematic model that incorporates all these motions would further enrich bionic robot locomotions and enhance their maneuverability.

In this paper, we proposed a general kinematic model capable of describing multiple biological motions and verified the effectiveness through a combination of simulation and experimentation using robotic fish as an example. The main contributions of this paper can be summarized as follows:

• A general kinematic model has been proposed that, by adjusting its parameters, allows the morphology to transition between diverse motions across a range of animals that rely on the body for movement, including but not limited to snakes, inchworms, octopuses, and fish. It is the most versatile kinematic model to date for describing multimodal locomotion of animals.
• By translating the general kinematic model into a motion control algorithm and combining it with virtual simulation, an efficient motion optimization framework for bionic robots with various design and actuation methods has been developed. This approach substantially simplifies the complexity of multimodal motion control in bioinspired robotic systems.

## Results

### General kinematic model and motion optimization framework

In this section, we present a comprehensive description of the general kinematic model and the motion optimization framework. The effectiveness of this approach is subsequently validated through robotic fish in the next section.

#### The general kinematic model

The model represents the curvature of the animal’s body midline and it can be expressed as

$$\kappa \left(s,\;t\right)=B_{1}+\left(c_{0}+c_{1}s+c_{2}s^{2}\right)\left[B_{2}+M\cos\left(\phi \left(t\right)-\mathrm{ks}\right)\right]$$ (1)

where s is the arc length measured along the body axis from the tip of the biological head, $c_{0}$, $c_{1}$, and $c_{2}$ are the amplitude envelope coefficient of curvature, $B_{1}$ is the first offset, $B_{2}$ is the second offset, M is the amplitude coefficient, and k is the body wave number (k=2π/λ, where λ is the body wavelength).

ϕ is the phase state that controls the properties of the model in the time dimension, and it can be described as

$$\overset{\cdot }{\phi }=\left[\frac{{\left(1+R\right)}^{2}}{4R}-\frac{R^{2}-1}{4R}\text{sign}\left(\sin\phi \right)\right]\omega$$ (2)

signλ=1,λ>00,λ=0−1,λ<0 (3)

where R is the time ratio between the restore phase and the beat phase, ω is the angular velocity, and the coefficient term of ω is an adjustable step function that can adjust its distribution of different segments within a cycle.

After constructing the curvature model, it is necessary to convert it into the coordinates of the centerline of the animal’s body. First, we need to calculate the angle of each element segment relative to the *x* axis

$$\theta \left(s,\;t\right)=\int_{0}^{s}\kappa \left(l,\;t\right)dl$$ (4)

Furthermore, the *x* and *y* coordinates at s can be obtained

$$x\left(s,\;t\right)=\int_{0}^{s}\cos\left(\theta \left(l,\;t\right)\right)dl$$ (5)

$$y\left(s,\;t\right)=\int_{0}^{s}\sin\left(\theta \left(l,\;t\right)\right)dl$$ 6)

#### Reproduce of different biological motions

**Fig. 2.**
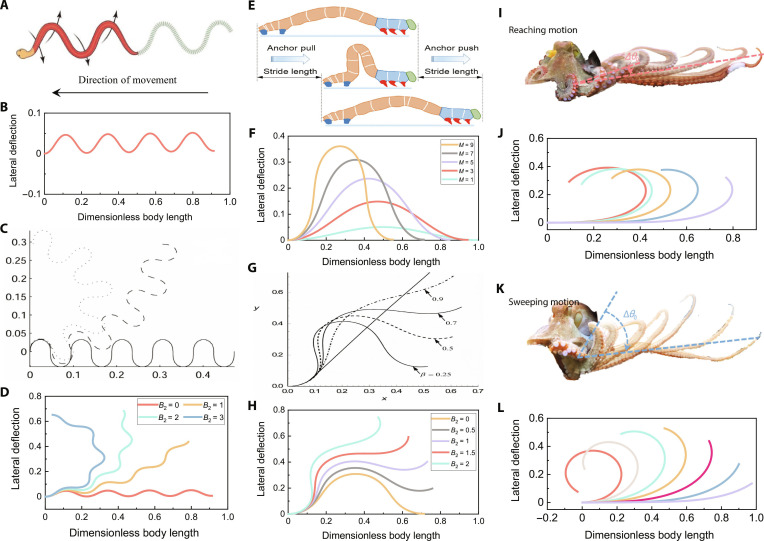
Movement of various animals. (A) Forward locomotion of snake [46]. (B) Imitation of the forward locomotion by the general kinematic model. (C) Turning locomotion generated by serpentine curve [36]. (D) Turning locomotion generated by the general kinematic model. (E) Crawling and climbing locomotion of inchworm [16]. (F) Imitation of the crawling and climbing locomotion by the general kinematic model. (G) Transitioning locomotion of inchworm generated by the model in [13]. (H) Imitation of the transitioning locomotion by the general kinematic model. Motion of octopus arm. (I) Reaching motion of octopus arm [17]. (J) Imitation of the reaching motion by the general kinematic model. (K) Sweeping motion of octopus arm [17]. (L) Imitation of the sweeping motion by the general kinematic model.

We take several biological objects mentioned in Introduction as examples and use the general kinematic model to reproduce their motion. The snake’s serpentine movement is illustrated in Fig. 2A, which utilizes the difference between normal and tangential friction to avoid sideslip and move forward. Several models that have been developed in previous studies to describe this movement are essentially based on body curvature [8,9,36]. Since our general kinematic model also uses body curvature as the dependent variable, it can be adapted to the serpentine curve through parameter adjustments. Specifically, by setting the amplitude envelope coefficients to $c_{0}=1,c_{1}=0,\text{ and }c_{2}=0$ and the remaining parameters $\left(M,\;B_{1},\;B_{2},\;k,\;R\right)$ to $\left(15,\;0,\;0,\;8\pi ,\;1\right)$, the model accurately captures the forward motion of snake, as shown in Fig. 2B. In addition to forward motion, the existing serpentine curve can also describe the motion of a snake during turns, as shown in Fig. 2C. Our model by adjusting the parameter $B_{2}$ based on the original parameters, can similarly achieve this effect, as illustrated in Fig. 2D.

Further, we analyze the motion of the inchworm. Despite the difference in plane orientation (horizontal and inclined), its crawling and climbing motions are essentially the same, both involving a “Ω”-shaped body curve for propulsion, as shown in Fig. 2E. To replicate these locomotions, we maintained the same amplitude envelope coefficients as those used for the serpentine curve and adjusted the parameters $\left(B_{1},\;B_{2},\;k,\;R\right)$ to $\left(0,\;0,\;2\pi ,\;1\right)$. By tuning M, the general kinematic model generates the corresponding motion, as illustrated in Fig. 2F. During the transition between crawling and climbing motions, the inchworm needs to cross planes, a process known as transitioning locomotion, as shown in Fig. 2G. By setting M to 7 and adjusting the value of B2, we can switch the model to the corresponding mode, as shown in Fig. 2H.

Next, we consider the soft arm of the octopus as an example. Its propagating motion can be categorized into 2 modes based on the propagation deflection angle: a straightforward point-to-point reaching motion and a large sweeping motion. The reaching motion, as shown in Fig. 2I, involves a small deflection angle for forward extension to reach the target point. To replicate this motion, we set the amplitude envelope coefficients in the model to $c_{0}=1,c_{1}=0.7,\text{and}\;c_{2}=-0.3$ and the other parameters to $\left(M,\;B_{1},\;B_{2},\;k,\;R\right)=\left(10,\;0,\;10,\;\pi ,\;1\right)$ , as shown in Fig. 2J. The sweeping motion, on the other hand, involves a larger deflection angle, thereby enlarging the path area of the arm, as illustrated in Fig. 2K. Through parameter adjustments, our general kinematic model can similarly describe the characteristics of this motion, as shown in Fig. 2L. The parameters were set as: $c_{0}=0.5,c_{1}=0.7,c_{2}=-0.3$, and $\left(M,\;B_{1},\;B_{2},\;k,\;R\right)=\left(4,\;0,\;4,\;\pi /2,\;1\right)$.

Overall, these mathematically explicit parameters carry universal physical interpretations across species. The amplitude envelope coefficients ($c_{1}=c_{2}=0$) physically dictate the spatial distribution of bending intensity along the animal’s body length. A constant envelope (governed solely by $c_{0}$) maintains a uniform bending amplitude from head to tail, facilitating steady-state rhythmic motions such as continuous slithering or cruising. Conversely, incorporating spatial terms ($c_{1}=c_{2}$) allows the model to locally amplify or attenuate the curvature. This is essential for capturing spatially varying dynamics, such as the increasingly pronounced propulsive tail beats of a swimming fish, or the highly localized, nonuniform grasping motions of an octopus arm. Meanwhile, the wave number k determines the spatial frequency of the bending nodes: A large k produces multiple simultaneous body waves characteristic of serpentine locomotion, while a small k restricts the body to fewer nodes, enabling large-scale, whole-body postural shifts such as an inchworm’s reaching or a fish’s C-turn. These examples collectively highlight the versatility of the general kinematic model in capturing animals’ motion. Researchers can customize the model to suit their specific bioinspired applications, ensuring alignment with the motion characteristics of their target animals.

#### Multimodal motion optimization framework

**Fig. 3.**
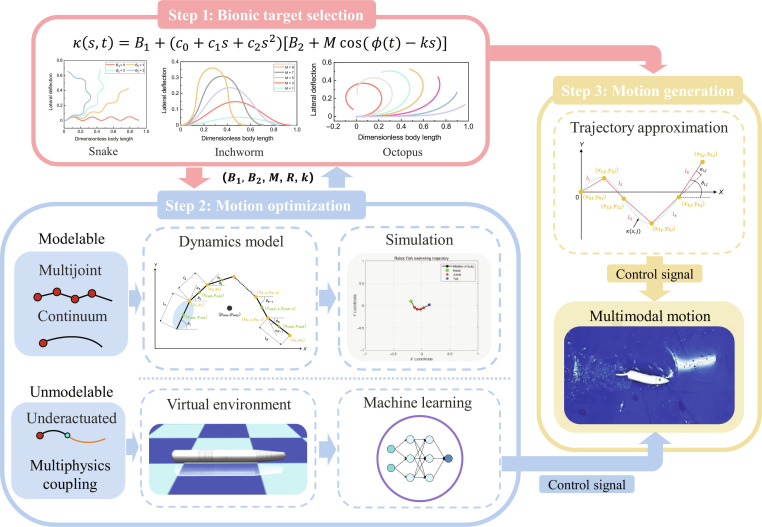
The motion optimization framework.

Through the general kinematics model, we can design diverse motions for bionic robots. However, predicting the specific performance of each motion remains challenging, and direct experimental validation on prototypes is often cost-prohibitive. To enhance the efficiency of motion design for bionic robots, we construct a multimodal motion optimization framework based on the general kinematic model. As illustrated in Fig. 3, the framework comprises 3 key steps:

• Bionic target selection. In this step, the boundary conditions of the general kinematic model need to be defined on the basis of the motion characteristics of the target animal, ensuring that its morphology closely matches that of the biological reference.
• Motion optimization. Subsequently, the morphology of the general kinematic model is further refined to ensure that the motion of the bionic robot aligns with the desired performance outcomes. Bionic robots utilize various actuation methods, among which multijoint and continuum-actuated systems are amenable to modeling. For example, Transeth et al. [37] developed a nonsmooth mathematical model for terrestrial multijoint robotic snakes. Boyer et al. [38] proposed a dynamic model for the swim motion of a continuous eel-like robot based on geometrically exact beam theory and Newton–Euler formulations. Chong et al. [39] extended the geometric mechanics framework to design contact patterns and verified its effectiveness in sidewinding limbless robots. Renda et al. [40] introduced a dynamics model for a general class of aquatic multibody, soft-structured robots using a Cosserat formalism. These modeling approaches are summarized in Table S1. Inspired by them, we can construct dynamic models tailored to the specific characteristics of a bionic robot prototype. By leveraging simulation results, the performance of the designed motion can be analyzed efficiently. This iterative process, facilitated by parameter adjustments in the general kinematic model, allows for continuous motion optimization, considerably reducing the development cycle for bionic robot motions.
• Motion generation. Once an ideal kinematic profile is obtained as a reference for motion design, the trajectory approximation method can be used to fit the model. This approach generates corresponding control signals, enabling the realization of multimodal locomotion in bionic robots.

However, the use of elastic materials in many bionic robots introduces important nonlinearities in their mechanical properties, making precise modeling a substantial challenge. For example, underactuated robotic systems with flexible, compliant segments are difficult to describe using traditional modeling techniques. To address this, our team [41] developed a deep-reinforcement-learning-based framework that successfully achieved online learning control for a bionic robotic eel with multiple passive structures. In addition, the complexity of real-world operational environments presents another barrier to accurate dynamic modeling. Fluid–structure interactions, in particular, introduce markedly uncertainties. To mitigate these challenges, Zhang et al. [42] integrated computational fluid dynamics with data-driven methodologies to construct a simulation environment. This framework, combined with deep reinforcement learning, enabled robotic fish to adapt and perform a variety of tasks. For robotic systems leveraging machine learning for motion control, the general kinematic model provides a robust foundation during motion training. This approach substantially reduces training demands while preserving motion diversity, addressing key issues such as lengthy training times and the risk of nonconvergent learning. Once an optimal motion is identified, it can be directly translated into control commands, enabling bionic robots to achieve versatile and efficient multimodal locomotion.

So far, we have completed the construction of the whole framework for multimodal motion optimization and control of different bioinspired robots. Following this framework, one can easily develop a bioinspired robot mimicking different biological objects and design its multimodal motion controller without much work.

### Validation of the proposed method with a multijoint robotic fish

To rigorously validate the proposed multimodal motion optimization framework (Fig. 3) in the physical world, we select the multijoint robotic fish as a representative hardware platform. In the following subsections, we systematically execute the 3 core steps of the framework: First, we perform a quantitative analysis to determine the kinematic boundary conditions for specific fish swimming modes; second, we construct a dynamic model to mathematically discretize and optimize these continuous motions in a simulation environment; finally, we deploy these optimized parameters as physical joint commands onto the robotic prototype for stationary and free-swimming experimental validations.

#### Quantitative analysis of fish locomotion

To analyze the characteristics of the model and determine the boundary conditions for describing different fish motions, we performed a quantitative analysis of various parameters. Based on experimental data observed by biologists [43,44], the amplitude envelope coefficients are set as follows: $c_{0}=1$, $c_{1}=-3.2$, and $c_{2}=5.6$. It should note that unless otherwise emphasized, all subsequent analyses in this paper use these envelope parameters.

From Eq. (1), it is evident that $B_{2}$ and M are coupled with the amplitude envelope, controlling the model’s offset and amplitude, respectively. Through their interaction, they can adjust the swing range of the model. We set $B_{1}=0$, M=1, k=π/2, ω=2π, and R=1 and vary $B_{2}$ to analyze its effect on the model’s limiting positions during reciprocating oscillation, as shown in Fig. S1. When $B_{2}=0$, the upper and lower bounds of the model are symmetric relative to the *x* axis, making it suitable for describing cruising-straight motion. When $0<B_{2}<M$, symmetry is broken, but the upper and lower bounds remain on either side of the *x* axis. This configuration results in a turning motion with low angular velocity, suitable for describing cruising-turn motions. When $B_{2}=M$, the lower bound of the model is nearly parallel to the *x* axis, enabling the model to rapidly change the fish’s forward direction, akin to the characteristics of a C-turn. When $B_{2}>M$, the model is completely biased toward one side of the *x* axis, which we define as a special C-turn, also referred to as a small-radius turn.

Since the influence of $B_{2}$ on the model is coupled with M and the amplitude envelope, it is not possible to independently adjust the curvature trend along the body length. To enable the model to exhibit the S-turn, which involves both positive and negative curvature, we introduce an independent bias $B_{1}$. One of the key differences between the S-turn and the cruising turn is that during the motion, the body is either completely biased toward one side of the *x* axis or, in the initial phase, nearly parallel to the *x* axis, meaning that the body curvature is consistently aligned from head to tail. To achieve this, we need to analyze the characteristics of the amplitude envelope, which is shown in Fig. S2. It can be seen that its minimum value occurs around 0.55. Thus, when $B_{2}=0$, the condition $\left|B_{1}\right|\geq \left|0.55\;M\right|$ must be met to realize this configuration. In addition to the initial curvature, the body wave number k is also a crucial factor influencing the S-turn. We set $B_{2}=0$, M=1, $B_{1}=0.55$, ω=2π, and R=1 and observe the trends of the kinematic model for different k. The model morphology at the same moment of the beat phase are compared in Fig. S3. When k<π/2, the curvature of the model is consistently aligned, exhibiting a C-turn-like moltion. When π/2≤k≤π, the curvature undergoes an inflection point, with the trend changing in the mid-section, resembling an S-turn. When k>π, the curvature trend changes multiple times, making the model unsuitable for describing the turning motions of fish.

**Fig. 4.**
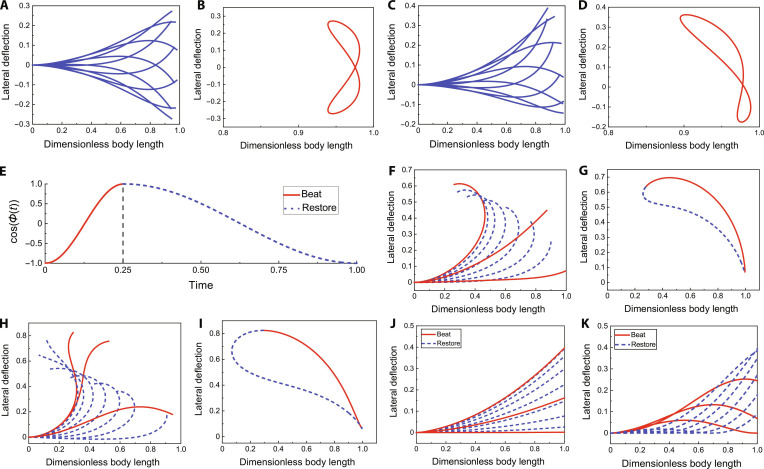
Various motion of the model (Movie S1). (A) Body curve of cruising straight. (B) The end trajectory of cruising straight. (C) Body curve of cruising turn. (D) The end trajectory of cruising turn. (E) Time domain properties of the model when R=3. (F) Body curve of C-turn. (G) The end trajectory of C-turn. (H) Body curve of S-turn. (I) The end trajectory of S-turn. (J) Body curve of C-turn in the previous model. (K) Body curve of S-turn in the previous model.

In addition to the parameters such as $B_{1}$ and $B_{2}$, which influence the model’s geometric morphology, the parameter R, which governs the temporal variation, is also crucial for describing the various motions of fish. In cruising, the fish’s motion is essentially evenly distributed in the time dimension, meaning that the durations of the beat and restore phases should be equal, i.e., R=1. For the description of cruising-straight motion, we set the model parameters $\left(B_{1},\;B_{2},\;M,\;\omega ,\;R,\;k\right)$ as $\left(0,\;0,\;1,\;2\pi ,\;1,\;\pi \right)$. The morphology of model is shown in Fig. 4A, with the trajectory of the end point shown in Fig. 4B. It is evident that the motion is perfectly symmetric relative to the *x* axis, resembling the typical traveling wave model used to describe cruising straight in fish. Keeping the other parameters unchanged, we modify the bias $B_{2}$ from 0 to 0.2, breaking the geometric symmetry of the model. This adjustment is well-suited for describing cruising-turn motion with lower turning velocities. The model morphology and the end-point trajectory are shown in Fig. 4C and D, respectively.

During fast turn, fish maximize their turning amplitude by rapidly curving their body and then gradually straightening it. Consequently, the time ratio between the restore phase and the beat phase $\left(t_{r}/t_{b}\right)$ should be greater than 1 (R>1). We set the period to 1 s and observed the temporal behavior of the model’s time characteristic, represented by $\cos\left(\phi \left(t\right)\right)$, when R=3, as shown in Fig. 4E. It can be observed that the duration of the beat phase is 0.25 s, while the duration of the restore phase is 0.75 s, yielding a time ratio $t_{r}/t_{b}=0.75/0.25=3$. This confirms the effect of R in adjusting the model’s time characteristics.

Based on this characteristic, we set the parameters $\left(B_{1},\;B_{2},\;M,\;\omega ,\;R,\;k\right)$ to $\left(0,\;1.5,\;1.5,\;2\pi ,\;3,\;\pi /2\right)$, transforming the model into the C-turn, as shown in Fig. 4F, with the end-point displacement shown in Fig. 4G. It can be observed that the model quickly curves into a large curvature C-shape during the beat phase and then gradually restores.

Furthermore, based on the previous analysis, for describing the S-turn, we set the parameters $\left(B_{1},\;B_{2},\;M,\;\omega ,\;R,\;k\right)$ to $\left(1.5,\;0,\;2.5,\;2\pi ,\;3,\;\pi \right)$. The model morphology and end-point displacement are shown in Fig. 4H and I. It can be seen that the model closely resembles the motion of a real fish during an S-turn.

In summary, the boundary conditions for generating various fish motions using this model are summarized in Table 1. By adjusting the parameters, the model not only simulates the cruising and small turning motions of fish but also overcomes the limitations of the previous model [32], where the independent variable *x* (the displacement along the head–tail axis) could only correspond to a unique solution for y (the lateral displacement of the body). The turning motions described by the previous model are shown in Fig. 4J and K. As seen in comparison with Fig. 4F and H, the bending is less pronounced, which prevents it from producing sharp fast-turn motions with large curvatures. This advancement further enriches the diversity of achievable motions.

**Table 1.** Boundary conditions to describe various fish motions of the model.

Motion	$B_{1}$	$B_{2}$	R	k
Cruising straight	$B_{1}=0$	$B_{2}=0$	R=1	k>0
Cruising turn	$B_{1}=0$	$0<\left|B_{2}\right|<|M|$	R=1	k>0
C-turn	$B_{1}=0$	$|B_{2}|\geq |M|$	R>1	0<k≤π/2
S-turn	$B_{1}\geq \text{Min}\left[\left|c_{0}+c_{1}s+c_{2}s^{2}\right|\right]\left|M\right|$	$B_{2}=0$	R>1	π/2<k≤π

#### Multijoint robotic fish

**Fig. 5.**
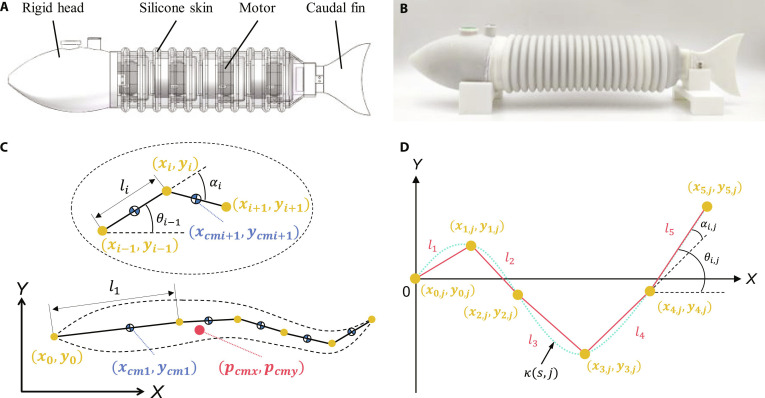
The overview of the robotics fish. (A) The computer-aided design (CAD) model of the robotics fish. (B) The prototype of the robotics fish. (C) Schematic illustration of coordinate systems and notations. (D) The discretized motion of the robotic fish.

To evaluate the practical effectiveness of the kinematic model in generating various motions, we developed a multijoint robotic fish as a verification platform, with its structure illustrated in Fig. 5A and B (see the “Design of multijoint robotic fish” section in Materials and Methods for details).

#### Dynamics modeling of robotic fish

Accurately modeling the dynamics of robotic fish is crucial for researchers to evaluate the effectiveness of different motions in simulation environments, markedly reducing the costs associated with design and optimization. Considering the characteristics of the robotic fish prototype, we simplify it as N=5 rigid links, assuming the center of mass of each link is located at its midpoint. The schematic illustration of coordinate systems and notations is shown in Fig. 5C. The detailed modeling process are provided in Supplementary Text.

To enable the dynamic model to execute the desired motion, the actuator torque of each joint must be continuously updated. This ensures that the links accurately follow our proposed kinematic model. First, we discretize the curvature in the time dimension as follows:

κsj=B1+c0+c1x+c2x2B2+McosϕTjU−ksj=1,…,U. (7)

where T represents the duration of a single cycle, the parameter U indicates the number of intervals the cycle is divided into, reflecting the temporal resolution of the control model, and j denotes the jth time oscillating sequence of the model.

Next, by combining the geometric features of the links with the general kinematic model Eq. (7), we can discretize the motion of the robotic fish (Fig. 5D) and calculate the coordinates of each joint:

xi,j−xi−1,j2+yi,j−yi−1,j2=li2xi,j∈∫0scos∫0sκljdldlyi,j∈∫0ssin∫0sκljdldli=1,…,N;j=1,…,U. (8)

where i indicates the ith link, j denotes the jth oscillating sequence, and N is the number of links. Although mapping the continuous curvature model to a discrete multijoint platform inherently introduces approximation errors, these deviations are maintained within an acceptable tolerance due to the robot’s adequate joint density.

Furthermore, based on the coordinate information, the angle of each link to the *x* axis and the rotation angle of each joint can be calculated:

$$\begin{array}{c} \theta_{i,j}=\text{arctan}\frac{y_{i,j}-y_{i-1,j}}{x_{i,j}-x_{i-1,j}}\;i=1,\ldots ,N;\;j=1,\ldots ,U. \end{array}$$ (9)

$$\alpha_{i,j}=\theta_{i,j}-\theta_{i-1,j}\;i=1,\ldots ,N;\;j=1,\ldots ,U.$$ (10)

Finally, a proportional-derivative controller is used to calculate the actuator torque of each joint required at different times:

$$t_{i}=K_{p,i}\left(\alpha_{i}^{*}-\alpha_{i}\right)-K_{d,i}{\overset{\cdot }{\alpha }}_{i}\;i=1,\ldots ,N.$$ (11)

where $K_{p,i}$ and $K_{d,i}$ are the gains of the controller, $\alpha_{i}^{*}$ is the target angle of each joint, and $\alpha_{i}$ is the current angle of each joint.

Using this dynamic model, we can efficiently evaluate the swimming performance of various motions in simulation and optimize the kinematic patterns of the robotic fish based on specific requirements (Movie S2). The hydrodynamic parameters in the simulation are set as follows: $C_{f}$ = 0.05 (drag coefficient in the tangential direction), $C_{D}$ = 1.5 (drag coefficient in the normal direction), $C_{M}$ = 1 (added inertia coefficient), and $C_{A}$ = 0 (neglecting added mass effects) [45]. The water density ρ is set to 1,000 kg/m^3^. For the proportional-derivative controller, the proportional and derivative gains $K_{p}$ and $K_{d}$ are set to 10 and 0.1, respectively.

**Fig. 6.**
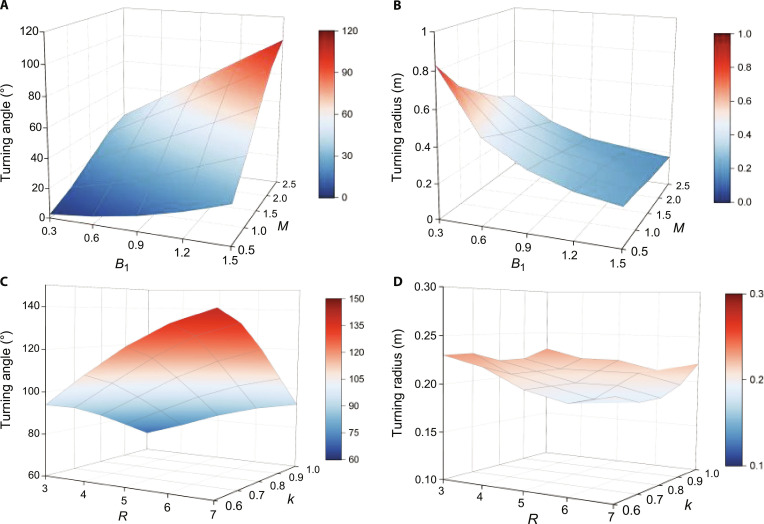
The influence of different parameters on the S-turn. (A) The influence of M and $B_{1}$ on turning angle in a single cycle. (B) The influence of M and $B_{1}$ on turning radius. (C) The influence of R and k on turning angle in a single cycle. (D) The influence of R and k on turning radius.

Taking the S-turn, the most complex motion in terms of parameters, as an example, we analyze its performance. As shown in Table 1, the key parameters influencing the S-turn morphology include $M,B_{1},R$, and k. We initially fix R=4 and k=π, then adjust M and $B_{1}$ to examine the resulting S-turn performance. Since M and $B_{1}$ directly influence the model’s curvature, increasing their values enhances body bending, leading to greater turning angles within a single cycle (Fig. 6A) and a reduced turning radius (Fig. 6B).

Further, we fix M=2 and $B_{1}=1.2$ and perform a quantitative analysis of R and k. The results, shown in Fig. 6C, indicate that as k increases within the range $\left(0.5\pi ,\;\pi \right]$, the turning angle of the S-turn gradually decreases. Conversely, increasing R amplifies the turning angle of the S-turn, as the shorter beat phase intensifies the interaction force between the fish body and the surrounding water. Note that R and k have little effect on the turning radius of the robotic fish (Fig. 6D), which can help us decouple to customize the motions.

#### Stationary tests

Having systematically evaluated and optimized the kinematic parameters in the virtual dynamic simulation, the subsequent crucial objective is to transition these theoretical findings into physical reality. To bridge this sim-to-real gap and validate the multimodal motion algorithm, we constructed an experimental platform for stationary tests (see the “Experimental setup of stationary tests” section in Materials and Methods for details). The experiment focused on evaluating the characteristics of 4 motions: cruising straight, cruising turn, C-turn, and S-turn. The motion parameters for each motion are presented in Table S2, and the snapshot sequences of the robotic fish’s body postures during these motions are shown in Fig. S4 and Movie S3. Distinct changes in body morphology were observed as the motions transitioned. During cruising-straight motion, the robotic fish maintained near symmetry along its central axis. In cruising-turn motion, the body exhibited a slight lateral shift. For both C-turn and S-turn motions, the fish successfully bent into the corresponding morphology, achieving curvatures considerably greater than those of the previous model [32]. These observed postures closely align with the theoretical trends predicted in Fig. 4, demonstrating that the designed algorithm effectively translates the general kinematic model into multimodal motion control signals.

#### Free swimming tests

To evaluate the swimming performance of various motions generated by the general kinematic model, we conducted a series of free-swimming tests using the robotic fish (Movie S4) and compared the experimental results with the dynamics simulation. The Experimental setup are shown in the “Experimental setup of free swimming tests” section in Materials and Methods for details.

**Fig. 7.**
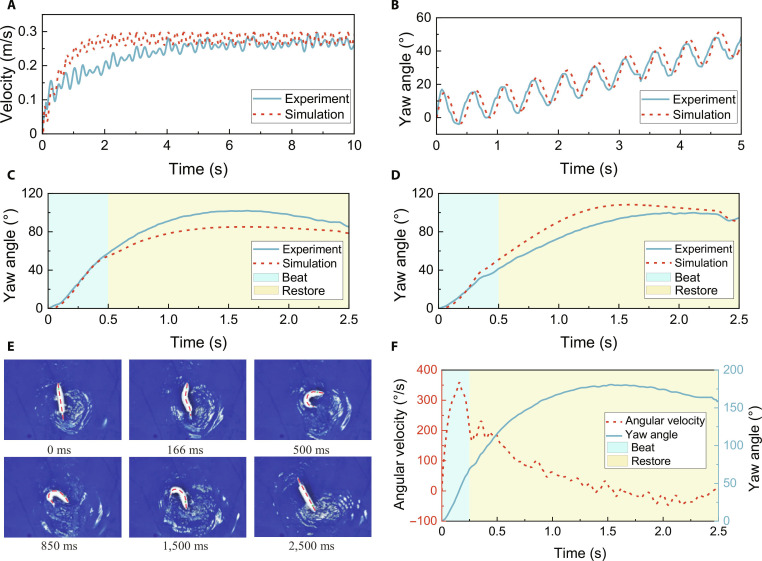
The swimming performance of robot fish. (A) Velocity of cruising straight. (B) Yaw angle of cruising turn. (C) Yaw angle of C-turn. (D) Yaw angle of S-turn. (E) Snapshot sequence of sharp S-turn.

The parameters used for different motions during the experiments are listed in Table S3. For cruising-straight motion, the driving frequency was set to 2 Hz, and the swimming velocity is shown in Fig. 7A. The results indicate that the robotic fish achieved a steady-state velocity faster in simulations than in experiments, yet both stabilized at approximately 0.275 m/s. This slight discrepancy in achieving steady-state velocity primarily stems from the simplified hydrodynamic modeling. The simulation utilizes constant hydrodynamic coefficients, which approximations cannot fully capture the complex, time-varying 3-dimensional (3D) fluid–structure interactions and vortex shedding present in the real fluid environment. During cruising-turn motion, with the same driving frequency of 2 Hz, the yaw angle variations are presented in Fig. 7B. The experimental and simulated results demonstrated high consistency. Within each motion cycle, the yaw angle exhibited periodic oscillations. Simultaneously, the robotic fish maintained a steady directional deviation, which is a key characteristic of cruising-turn motion. For C-turn and S-turn motions, the driving frequency was reduced to 0.4 Hz, corresponding to a motion period of 2.5 s. The yaw angle variations for these motions are shown in Fig. 7C and D, respectively, demonstrating excellent agreement between simulation and experiment. With R=4, the first 0.5 s of each cycle represents the beat phase, during which the robotic fish rapidly bends its body, causing a sharp change in yaw angle. The remaining 2 s correspond to the restore phase, where the body gradually straightens, generating counteracting momentum. Due to rotational inertia, the yaw angle initially continues to increase before gradually decreasing. Notably, the C-turn motion reached a peak yaw angle of 100° more rapidly than the S-turn motion, although both ultimately achieved a 90° directional change.

These results collectively confirm that the proposed general kinematic model successfully enables the robotic fish to perform a wide variety of swimming motions, while validating the accuracy of the constructed dynamic model and simulation methodology. This methodological framework facilitates rapid motion optimization and precise motion control for robotic fish. Building on the quantitative analysis of S-turn parameters in Fig. 6, it was found that increasing $M,B_{1},$ and R, while decreasing k, within certain ranges enhances the turning angle per cycle. To improve S-turn performance, we set $M=2.5,B_{1}=1.5,B_{2}=0,R=9,$ and k=0.6, with a driving frequency of 0.4 Hz. The snapshot sequence of the resulting turning motion is shown in Fig. 7E (Movie S5), demonstrating a large-angle directional change. According to measurements in Kinovea software, its turning radius is 0.19 body length (8.9 cm). The yaw angle and angular velocity during this cycle are shown in Fig. 7F, where the peak instantaneous angular velocity reached 350°/s and the final turning angle was 160°. These findings validate the effectiveness of the simulation-based optimization strategy and highlight the potential of the proposed general kinematic model for describing sharp fast turn motions in fish.

## Conclusion

This study proposes a general kinematic model that integrates the animal’s body curvature equation with a nonlinear oscillator to illustrate the diverse motion of animals, including but not limited to fish, snakes, inchworms, and octopuses. By coupling it with the dynamic model or machine learning, a motion optimization framework has been constructed, which substantially simplifies the complexity of multimodal motion control for bionic robots. Taking the robotic fish as an example, we have efficiently realized motions such as cruising-straight, cruising-turn, and various fast turn motions, especially the sharp fast turn with a large turning angle and high angular velocity. To the best of our knowledge, this represents the most versatile kinematic model for characterizing animal motions to date. Traditional fish traveling wave equations and specific snake curves are typically restricted to symmetric cruising or specific rhythmic undulations, struggling to describe asymmetric, large-curvature maneuvers. Conversely, standard continuum robot models excel in static spatial bending but lack the intrinsic dynamic temporal rhythms required for continuous locomotion. Our framework bridges these gaps by seamlessly integrating large-curvature spatial deformations with dynamic rhythmic generation, unifying these diverse locomotion modes into a single comprehensive framework.

To quantitatively benchmark the proposed framework, a detailed comparison of the turning performance against various state-of-the-art control methods based on multijoint robotic fish platforms is presented in Table S5. As can be seen, the proposed method exhibits a pronounced advantage in terms of turning radius, enabling nearly in-place turning maneuvers (Movie S5). Both the peak angular velocity and the turning angle also outperform the majority of existing methods. Moreover, the turning motion can be generated simply by adjusting the model parameters, without the need for carefully handcrafted motion patterns, resulting in low control complexity and high practicality for engineering applications. These results validate the effectiveness of the proposed framework and highlight its potential as a general and practical paradigm for modeling, optimization, and control of bioinspired robotic locomotion.

In future work, we will first explore methods to extend the proposed general kinematic model to 3D locomotion. Theoretically, this can be achieved by introducing orthogonal curvature components and coupling pitch and roll parameters to enable complex 3D maneuvers such as diving or spiraling. Besides, we will systematically explore the parameter space for diverse biological motions and use learning-based techniques to holistically optimize these model parameters (including amplitude envelope coefficients). This optimization will target specific practical requirements, such as maximizing energy efficiency. Furthermore, while the physical validation in this study is representatively conducted on a robotic fish, the proposed framework exhibits exceptional scalability to more complex morphologies. For soft-bodied systems, its intrinsic continuous spatial curvature functions can be directly mapped to soft actuators, bypassing the discrete joint approximations required for rigid robots. For multilimbed platforms (e.g., quadrupedal or salamander-like robots), the model can be scaled to serve as a central spinal generator; coupling this continuous torso undulation with limb end-effectors will effectively coordinate gait phases, extend stride length, and enhance turning agility. Deploying this universal framework across these diverse physical prototypes remains a primary focus of our ongoing research. Finally, we plan to enhance the biomimetic degree of motion by coupling our kinematic framework with advanced actuators that inherently possess fish-muscle-like characteristics (e.g., variable stiffness and passive compliance), thereby bridging the gap between kinematic abstraction and true biomechanical fidelity.

Looking beyond these immediate biorobotic enhancements, the proposed framework holds important potential for broader interdisciplinary applications. In adaptive control, the model’s explicit and decoupled parameters facilitate real-time motion tuning, enabling autonomous systems to quickly adapt to unpredictable environmental perturbations. For exploration robotics, the framework’s capability to seamlessly generate diverse, large-curvature maneuvers equips robots with the extreme agility required for unstructured environments. By translating these mathematically explicit curves into complex obstacle avoidance trajectories, the model enables continuous exploration in highly confined spaces. In the biomedical domain, the framework’s ability to generate continuous, biomimetic rhythmic curves offers promising implications for clinical rehabilitation devices. Mapping these smooth, biologically inspired profiles onto soft exoskeletons or continuous passive motion machines could foster safer and highly compliant human–robot interactions during motor recovery therapies.

## Materials and Methods

### Design of multijoint robotic fish

The rigid head is engineered to house the core electronic components, functioning as the central processing and control hub. This compact compartment accommodates a microcontroller unit, a rechargeable battery, a wireless communication module, and an inertial measurement unit, collectively acting as the “brain” of the system. These components enable real-time sensing, command transmission, and bidirectional communication, forming the foundation for intelligent motion control. The body of the robotic fish features a modular design comprising 4 serially connected motors. Each motor represents an articulating joint, culminating in a hard caudal fin affixed to the terminal segment. By orchestrating the coordinated actuation of the motors, the robotic fish can execute a wide range of motions, including those requiring sharp maneuvers. In addition, silicone skin envelops the body of the robotic fish, providing waterproofing and enhancing the system’s hydrodynamic performance. The technical specifications of the robotic fish are detailed in Table S4.

### Experimental setup of stationary tests

The experimental platform is depicted in Fig. S5. The robotic fish was secured in water using clamps attached to fixed aluminum profiles, and a 60-fps camera was positioned directly above the fish to capture close-up observations of its motions. Parameter U in Eq. (7) was set to 100, discretizing each motion cycle into 100 frames for precise control.

### Experimental setup of free swimming tests

The experiments were carried out in a water tank measuring 4 m in length, 3 m in width, and 0.6 m in depth. A 60-fps camera system positioned above the tank captured the swimming trajectories of the robotic fish (Fig. S6). Additional data, such as swimming velocity and yaw angle, were acquired through an onboard inertial measurement unit sensor and postprocessing of the video by Kinovea software.

## Data Availability

The data that support the findings of this study are available from the corresponding authors upon reasonable request.

## Supplementary Materials

Supplementary 1 Supplementary Text Figs. S1 to S6 Tables S1 to S5 Movies S1 to S5 References [47–56]
