# Supplementary material for: A General Kinematic Model for Multimodal Locomotion in Bioinspired Robots
Source: Research (Wash D C). 2026 May 4;9:1275. doi: 10.34133/research.1275 (PMC13136628; doi:10.34133/research.1275)
Supplement: Supplementary 1 — Supplementary Text Figs. S1 to S6 Tables S1 to S5 Movies S1 to S5 References [47–56] [file research.1275.f1.zip › Supplementary Materials.docx]

Supplementary Materials

**A General Kinematic Model for Multi-Modal Locomotion in Bio-Inspired Robots**

Zicun Hong^1,2^, Junwen Fei^1^, Weihua Li^1^, Jun Yan^3^, Junzhi Yu^4^, Feng Tian^2^, and Yong Zhong^1*^

^1^ Shien-Ming Wu School of Intelligent Engineering, South China University of Technology

^2^ GRG Banking Equipment Co., Ltd.

^3^ Chinese Institute of Marine & Offshore (ZH) Co., Ltd.

^4^ State Key Laboratory for Turbulence and Complex Systems, Department of Advanced Manufacturing and Robotics, College of Engineering, Peking University

^*^Address correspondence to: zhongyong@scut.edu.cn

**The PDF file includes:**

Supplementary Text

Fig. S1 to S6

Table S1 to S5

**Other Supplementary Materials includes the following:**

Movie S1 to S5

**Supplementary Text. Detailed process of dynamics modeling**

In this section we briefly outline the core parts of our model, more details can be found in (*32*). As shown in Fig. 5C, $(x_{i}{,y}_{i})$ represents the endpoint of the $i$th link, $(x_{cmi}{,y}_{cmi})$ represents the center of mass (CM) of the $i$th link, and $(P_{cmx}{,P}_{cmy})$ represents the CM of the whole robotic fish. Vectors $\boldsymbol{\theta}=\left[ \theta_{1},...,\theta_{N} \right]^{T}\in\mathbb{R}^{N}$ and $\boldsymbol{\alpha}=\left[ \alpha_{1},...,\alpha_{N-1} \right]^{T}\in\mathbb{R}^{N-1}$ are the aggregate of the link angle and joint angle respectively.

During the calculation process, each link is simplified as an elliptic cylinder, described by the following vectors. Major radius: $\mathbf{a}$ = diag[0.035, 0.035,0.035, 0.035,0.035] m, minor radius: $\mathbf{b}=\mathrm{diag}\left[ 0.035, 0.035, 0.035, 0.035, 0.005 \right] m$, length: $\mathbf{l}$ = diag[0.155, 0.062, 0.062, 0.062, 0.122] m, mass: $\mathbf{m}$ = diag[0.45, 0.25, 0.25, 0.25, 0.05]$\mathrm{kg}$, and moment of inertia: $\mathbf{J}=\frac{\mathbf{1}}{\mathbf{3}}\mathbf{m}\mathbf{l}^{\mathbf{2}}kg\cdot m^{2}$.

The acceleration of the CM of the whole robotic fish can be expressed as

$\left[ \begin{aligned} \ddot{P}_{cmx} \\ \ddot{P}_{cmy} \end{aligned} \right]=\frac{1}{M}\left[ \begin{matrix} \boldsymbol{e}^{\boldsymbol{T}} & \boldsymbol{0} \\ \boldsymbol{0} & \boldsymbol{e}^{\boldsymbol{T}} \end{matrix} \right]\left[ \begin{matrix} \boldsymbol{f}_{x} \\ \boldsymbol{f}_{y} \end{matrix} \right]$ (S1)

where $M$ is the mass of the robotic fish, $\mathbf{e}=\left[ \begin{matrix} 1, & \ldots, & 1 \end{matrix} \right]^{T}\in\mathbb{R}^{N}$, and $\mathbf{f}={[f_{x},f_{y}]}^{T}\in\mathbb{R}^{N}$ is the fluid forces acting on links which can be expressed as

$\mathbf{f}\boldsymbol{=}\left[ \begin{matrix} \mathbf{f}_{\mathbf{x}} \\ \mathbf{f}_{\boldsymbol{y}} \end{matrix} \right]=\left[ \begin{matrix} \mathbf{f}_{\mathbf{A}_{\mathbf{x}}} \\ \mathbf{f}_{\mathbf{A}_{\mathbf{y}}} \end{matrix} \right]+\left[ \begin{matrix} \mathbf{f}_{\mathbf{D}_{\mathbf{x}}} \\ \mathbf{f}_{\mathbf{D}_{\mathbf{y}}} \end{matrix} \right]$ (S2)

where the vectors $\mathbf{f}_{\mathbf{A}_{\mathbf{x}}}$ and $\mathbf{f}_{\mathbf{A}_{\mathbf{y}}}$ represent the added mass effects and vectors $\mathbf{f}_{\mathbf{D}_{\mathbf{x}}}$ and $\mathbf{f}_{\mathbf{D}_{\mathbf{y}}}$ represent the drag forces.

We use the motion state of the CM of each link to calculate the fluid forces, and the acceleration can be expressed as

${\ddot{\mathbf{X}}}_{\mathrm{cm}}=\mathbf{T}^{-1}\left[ \begin{aligned} \mathbf{A}\left( {\frac{\mathbf{l}}{2}\mathbf{C}}_{\theta}{\dot{\boldsymbol{\theta}}}^{\boldsymbol{2}}\boldsymbol{+}\frac{\mathbf{l}}{2}\mathbf{S}_{\theta}\ddot{\boldsymbol{\theta}} \right) \\ \ddot{P}_{\mathrm{cmx}} \end{aligned} \right]\in\mathbb{R}^{N\times1}$ (S3)

${\ddot{\mathbf{Y}}}_{\mathrm{cm}}=\mathbf{T}^{-1}\left[ \begin{aligned} \mathbf{A}\left( {\frac{\mathbf{l}}{2}\mathbf{S}}_{\theta}{\dot{\boldsymbol{\theta}}}^{\boldsymbol{2}}\boldsymbol{-}{\frac{\mathbf{l}}{2}\mathbf{C}}_{\theta}\ddot{\boldsymbol{\theta}} \right) \\ \ddot{P}_{\mathrm{cmy}} \end{aligned} \right]\in\mathbb{R}^{N\times1}$ (S4)

$\mathbf{T}=\left[ \begin{aligned} \mathbf{D} \\ \frac{1}{M}\mathbf{m} \end{aligned} \right]\mathbb{\in R}^{N\times N}$ (S5)

where $\mathbf{A}$ and $\mathbf{D}$ represent an addition matrix and a difference matrix, respectively, which are used for matrix operation. $\ddot{\boldsymbol{\theta}}$ is the angular acceleration of the link relative to the x-axis in the global coordinate system, which can be calculated by

$\ddot{\boldsymbol{\theta}}\boldsymbol{=}\mathbf{D}^{T}\mathbf{t}\boldsymbol{-}\mathbf{l}\mathbf{S}_{\theta}\mathbf{A}^{T}\mathbf{h}_{\mathbf{x}}\boldsymbol{+}\mathbf{l}\mathbf{C}_{\theta}\mathbf{A}^{T}\mathbf{h}_{\mathbf{y}}\boldsymbol{+}\boldsymbol{\tau}$ (S6)

where $\mathbf{S}_{\theta}=diag(sin\boldsymbol{\theta})\mathbb{R}^{N\times N}$, $\mathbf{C}_{\theta}=diag(cos\boldsymbol{\theta})\mathbb{R}^{N\times N}$, vector $\mathbf{t}={[t_{1},\cdots,t_{N}]}^{T}\in\mathbb{R}^{N-1}$ is the actuator torque of each joint, $\text{h=}{\text{[}\text{h}_{\text{x}}\text{,}\text{h}_{\text{y}}\text{]}}^{\text{T}}\text{∈}\mathbb{R}^{\text{N-1}}$ represents the joint constraint force, and $\boldsymbol{\tau}$ is fluid torque acting on the link, which can be expressed as

$\boldsymbol{\tau}\boldsymbol{=-}\boldsymbol{\lambda}_{\boldsymbol{1}}\ddot{\boldsymbol{\theta}}\boldsymbol{-}\boldsymbol{\lambda}_{\boldsymbol{2}}\dot{\boldsymbol{\theta}}\boldsymbol{-}\boldsymbol{\lambda}_{\boldsymbol{3}}\mathrm{diag}\left( sgn(\dot{\boldsymbol{\theta}}) \right){\dot{\boldsymbol{\theta}}}^{\mathbf{2}}$ (S7)

where vector $\boldsymbol{\lambda}_{\mathbf{1}}$, $\boldsymbol{\lambda}_{\mathbf{2}}$, $\boldsymbol{\lambda}_{\mathbf{3}}$ depend on the fluid characteristics and the shape of the robotic fish. The calculation method can be referred to (*47, 48*).


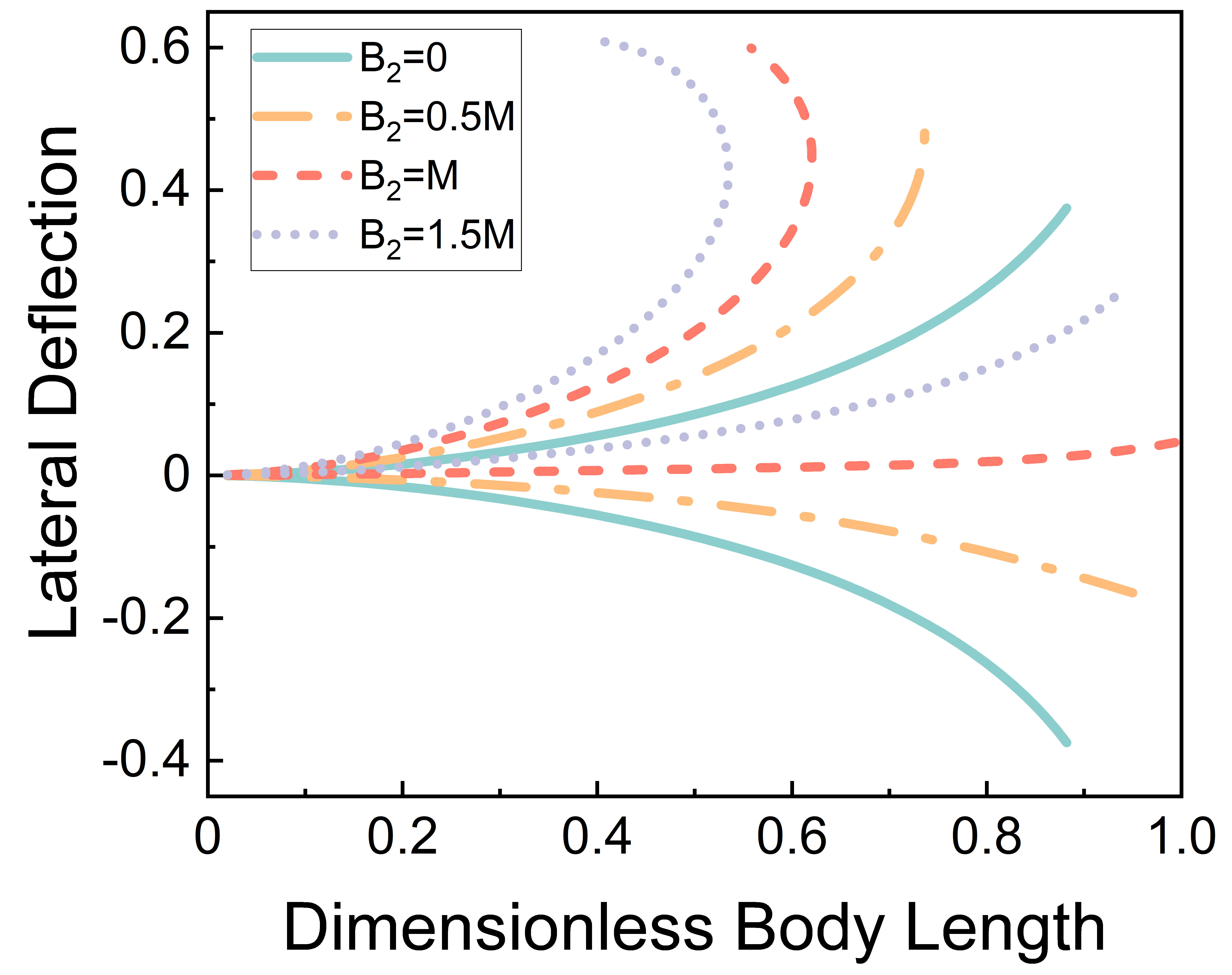


**Fig. S1.** The influence of $B_{2}$ and $M$ on the oscillation limit position of the model.

**Fig. S2.** The trajectory of the envelope ($c_{0}=1$， $c_{1}=-3.2$，$c_{2}=5.6$).


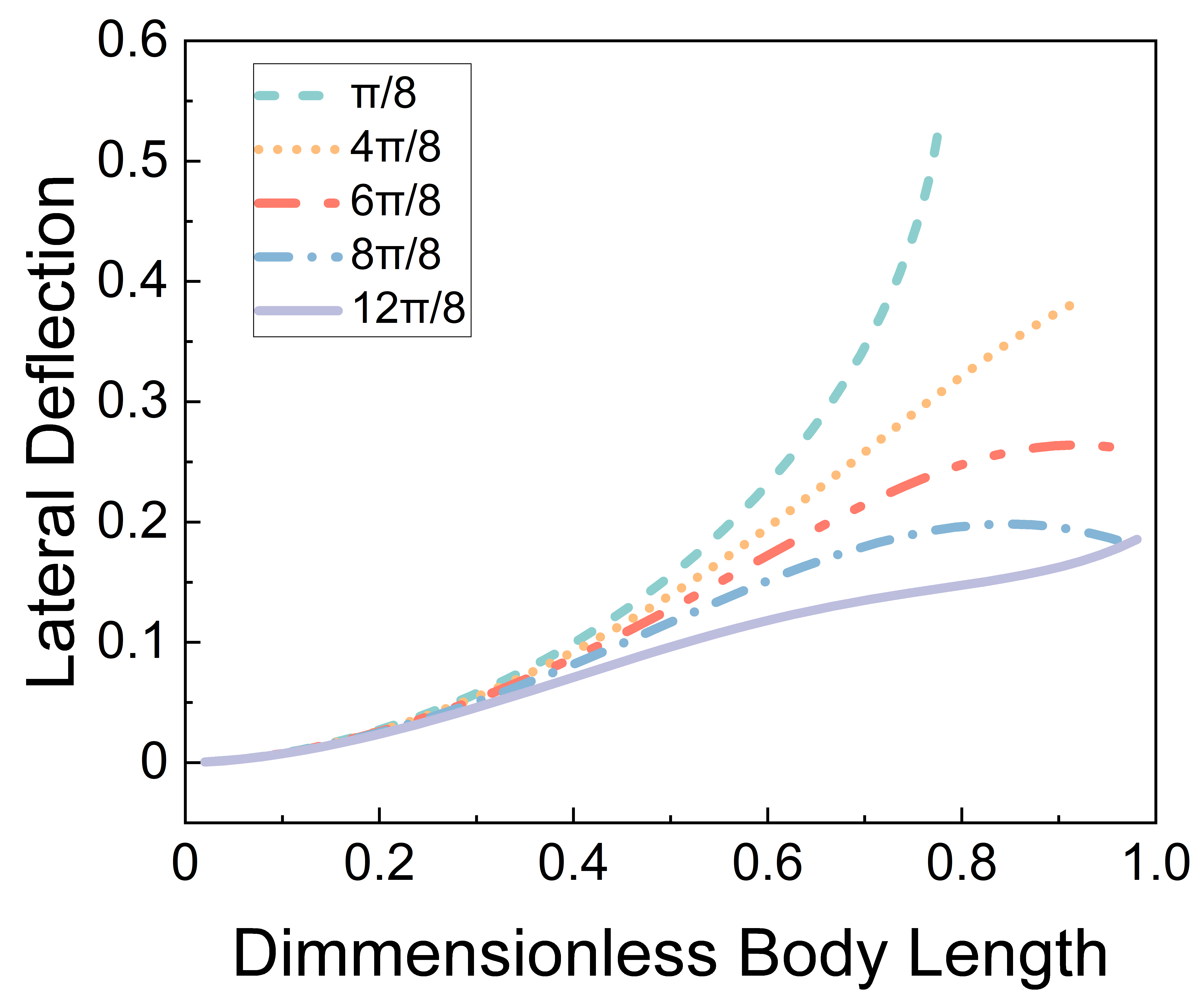


**Fig. S3.** The influence of $k$ on the model.


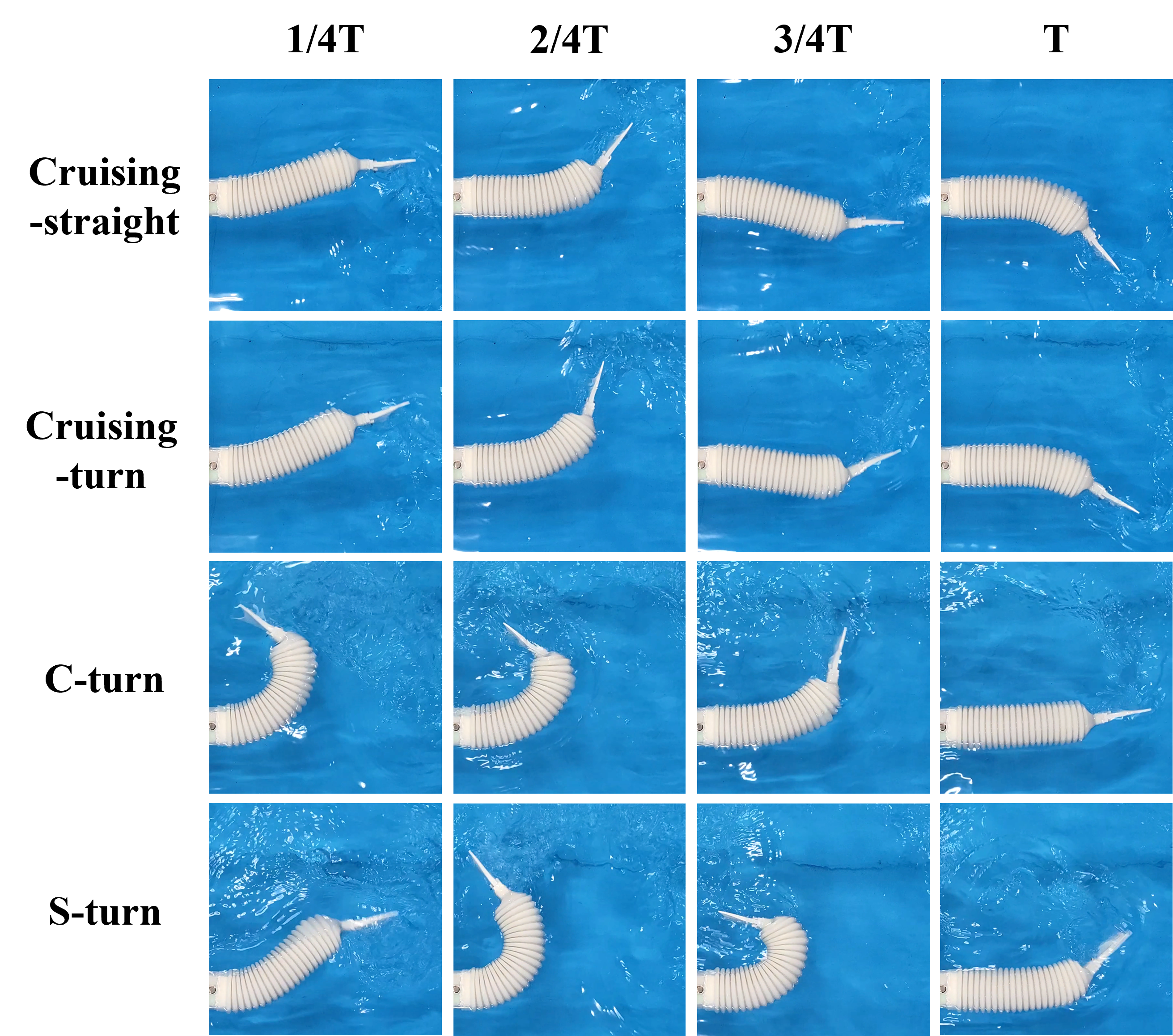


**Fig. S4.** Snapshot sequence of the robotic fish body posture with different swimming motions.

**Fig. S5.** Experimental platform of stationary tests.

**Fig. S6.** Experimental platform of free swimming tests.

**Table S1**. Modeling methods for bionic robots with different actuator types.

| **Actuator Type** | **Environment** | **Method** | **References** |
| --- | --- | --- | --- |
| Multi-joint | Land | Newton–Euler | 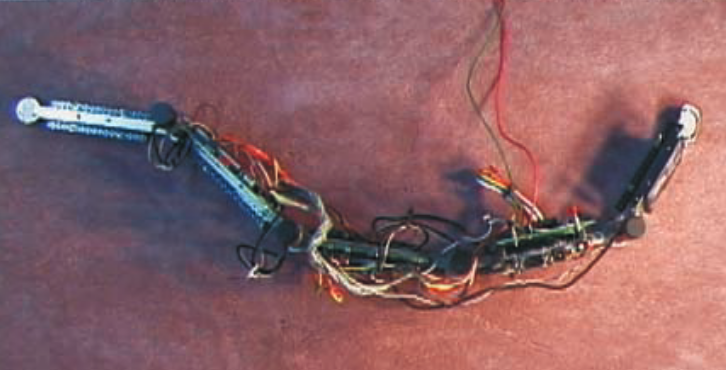 (*36*) 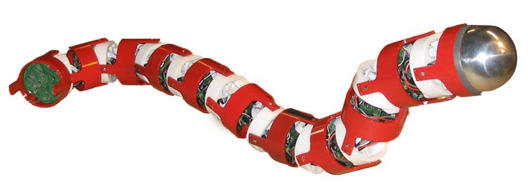 (*38*) 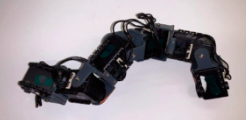 (*40*) |
|  |  | Lagrange | 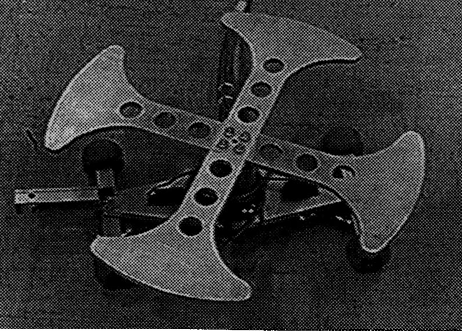 (*49*) |
|  | Water | Newton–Euler | 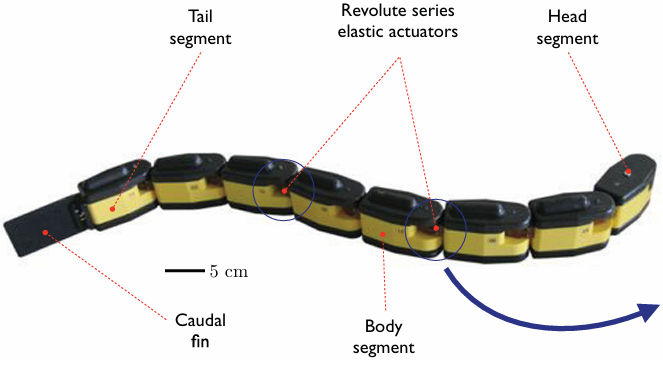 (*50*) 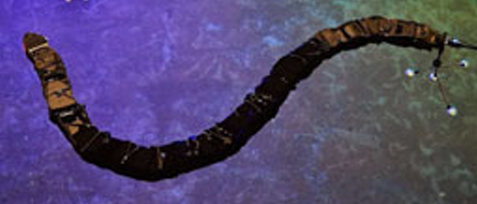 (*48*) |
|  |  | Lagrange | 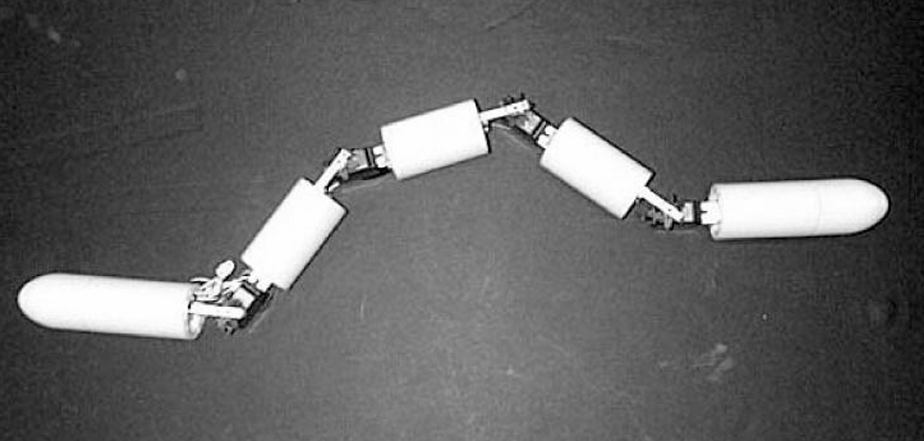 (*51*) |
| Continuum | Land | Newton–Euler | 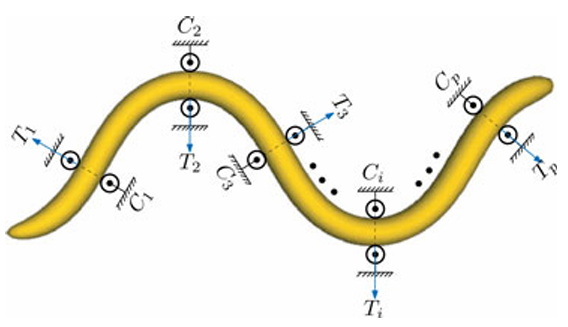 (*52*) 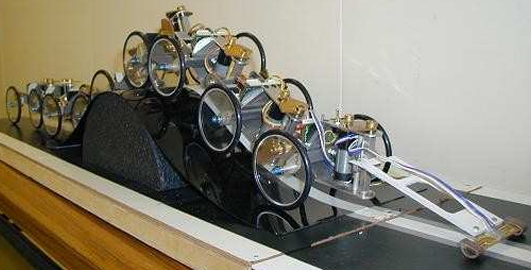 (*53*) |
|  | Water | Newton–Euler | 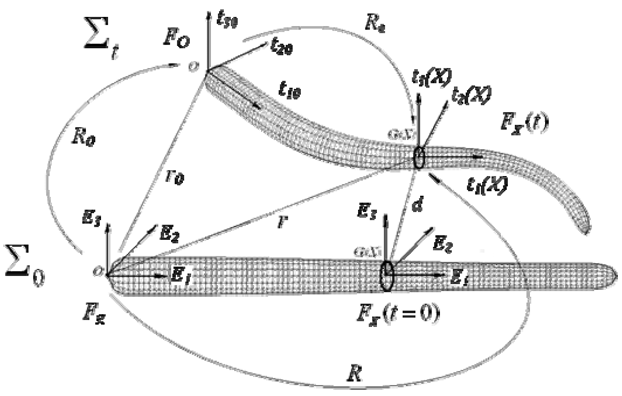 (*39*) 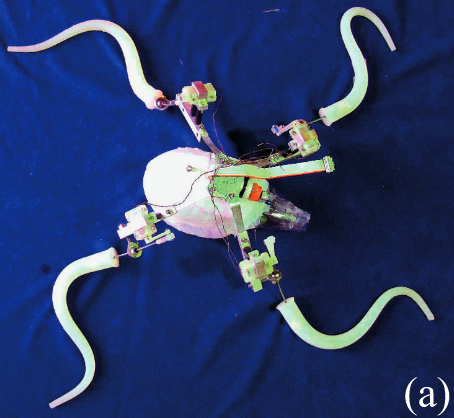 (*41*) 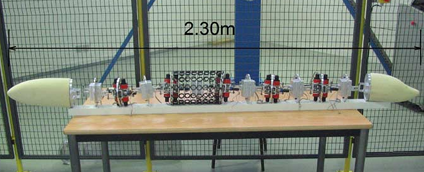 (*54*) |

**Table S2.** Parameter settings for different motions in stationary tests.

| **Motion** | $\boldsymbol{M}$ | $\boldsymbol{B}_{\boldsymbol{1}}$ | $\boldsymbol{B}_{\boldsymbol{2}}$ | $\boldsymbol{R}$ | $\boldsymbol{k}$ |
| --- | --- | --- | --- | --- | --- |
| Cruising-straight | 1.5 | 0 | 0 | 1 | $\pi$ |
| Cruising-turn | 1.5 | 0 | 0.4 | 1 | $\pi$ |
| C-turn | 1.5 | 0 | 1.5 | 3 | $\pi/2$ |
| S-turn | 2.5 | 1.5 | 0 | 3 | $\pi$ |

**Table S3.** Parameter settings for different motions in free swimming tests.

| **Motion** | $\boldsymbol{M}$ | $\boldsymbol{B}_{\boldsymbol{1}}$ | $\boldsymbol{B}_{\boldsymbol{2}}$ | $\boldsymbol{R}$ | $\boldsymbol{k}$ |
| --- | --- | --- | --- | --- | --- |
| Cruising-straight | 1 | 0 | 0 | 1 | $\pi$ |
| Cruising-turn | 1 | 0 | 0.2 | 1 | $\pi$ |
| C-turn | 1.5 | 0 | 1.5 | 4 | $\pi/2$ |
| S-turn | 2.5 | 1.5 | 0 | 4 | $\pi$ |

**Table S4**. Technical parameters of robotic fish.

| **Items** | **Specifications** |
| --- | --- |
| Dimension | ~ 463mm(L) × 72mm(W) × 98mm(H) |
| Mass | ~ 1.25 kg |
| MCU | STM32F407 |
| Motor | Yeahbot ZX15D |
| Battery | 7.4V Li Battery |
| Communication | RF 433M |
| IMU | IMU JY61 |

**Table S5**. Comparison of turning performance of different control methods.

| **Method** | **Turning radius**  **(BL)** | **Peak angular velocity**  **(°/s)** | **Turning angle**  **(°/cycle)** | **Complexity** |
| --- | --- | --- | --- | --- |
| (*34*) | 0.3 | 670 | 213 | High |
| (*25*) | 0.3 | 120 | 110 | Medium |
| (*55*) | 0.52 | 51 | / | Low |
| (*56*) | / | 130.5 | 80 | High |
| Our method | 0.19 | 350 | 160 | Low |
